# Statistical learning in domestic chicks is modulated by strain and sex

**DOI:** 10.1038/s41598-020-72090-8

**Published:** 2020-09-15

**Authors:** Chiara Santolin, Orsola Rosa-Salva, Bastien S. Lemaire, Lucia Regolin, Giorgio Vallortigara

**Affiliations:** 1 Center for Brain and Cognition, Universitat Pompeu Fabra, Barcelona, Spain; 2 Centre for Mind/Brain Sciences, University of Trento, Rovereto, Italy; 3 Department of General Psychology, University of Padova, Padua, Italy

**Keywords:** Psychology, Neuroscience

## Abstract

Statistical learning is a key mechanism for detecting regularities from a variety of sensory inputs. Precocial newborn domestic chicks provide an excellent model for (1) exploring unsupervised forms of statistical learning in a comparative perspective, and (2) elucidating the ecological function of statistical learning using imprinting procedures. Here we investigated the role of the sex of the chicks in modulating the direction of preference (for familiarity or novelty) in a visual statistical learning task already employed with chicks and human infants. Using both automated tracking and direct human coding, we confirmed chicks’ capacity to recognize the presence of a statistically defined structure underlying a continuous stream of shapes. Using a different chicken strain than previous studies, we were also able to highlight sex differences in chicks’ propensity to approach the familiar or novel sequence. This could also explain a previous failure to reveal statistical learning in chicks which sex was however not determined. Our study confirms chicks’ ability to track visual statistics. The pivotal role of sex in determining familiarity or novelty preferences in this species and the interaction with the animals’ strain highlight the importance to contextualize comparative research within the ecology of each species.

## Introduction

Extracting regularities from sensory inputs is a core capacity for effective adaptation in human and non-human species. Over the past 20 years, a substantial body of research has pointed to statistical learning as a key mechanism. By tracking regularities from a variety of sensory inputs, statistical learning mechanisms build the foundation for further perceptual and cognitive processes. The general nature of statistical learning has been shown across modalities, tasks and species, pointing to a powerful, yet constrained, learning process^1–4^.

In the present paper, we focus on a specific statistical learning ability available across species: the processing of structured *visual* inputs. Human infants are sensitive to distinct types of regularities in the visual domain. Statistical learning in infants has been shown for both temporal and spatial inputs, such as streams of shapes or multi-element displays^5–11^. Such mechanism seems to be spontaneous, rapid and available from birth, allowing infants to extract relevant patterns for further processing of visual scenes^4^. Several non-human species have revealed similar visual statistical learning abilities. Non-human primates can learn visual structures of different complexity, such as perceptual dependencies between string elements^12^, ordering patterns generated by artificial grammars^13,14^, hierarchical structures^15,16^, and orthographic patterns^17^. Evidence has also been reported for avian species using both spatial configurations and temporal sequences, such as orthographic patterns^18^ and artificial grammars involving visual elements^19^. Precocial avian species^20,21^ provide an excellent model to study statistical learning in a comparative perspective to human infants. Newly-hatched domestic chicks have shown similar capacities to young infants. Using conditioning methods, chicks have been shown to track spatial relations among visual items following ABA or AAB patterns^22^ similar to Saffran et al.^23^.

Chicks of precocial avian species present a unique advantage for the comparative investigation of statistical learning. Thanks to the prominent learning mechanism of filial imprinting, species as domestic chicks (*Gallus gallus*) offer the opportunity to study unsupervised forms of statistical learning, like it is done in human adults and infants. Using imprinting procedures, it has been possible to demonstrate spontaneous sensitivity to spatial arrangements of visual and audiovisual displays^24–27^. However, chicks’ implicit learning capabilities are not limited to spatial regularities. In our previous work^28^, we showed spontaneous sensitivity to the temporal statistics of a sequence of visual items. In this study, we adapted a paradigm initially developed for human infants^6,9^. Newborn chicks detected the probabilistic structure (co-occurrence frequencies or transitional probabilities) defining the orderings of a visual stream. This very first evidence showed that at birth, and without any reward, chicks were immediately tuned to regularities characterizing the visual environment. Our results also expanded prior infant evidence showing limited visual statistical learning abilities at birth. Indeed, chicks revealed even superior abilities than human infants, succeeding in the task also when sequences were composed of up to six elements^9,28^. This may be due to biological constraints on visual processing in human newborns, while the maturation of chicks’ visual system is already advanced at birth^29–31^.

Research on statistical learning in chicks has thus a great comparative significance in relation to infant studies. However, when working with animal models it is of paramount importance to contextualize research within the ecology of each species. We must thus identify the adaptive function of the statistical learning mechanisms discovered in chicks. To do so, we must consider this in the context of the filial imprinting mechanism. This powerful form of learning by exposure causes chicks to restrict their social approach towards the first conspicuous object(s) they are exposed to for a sufficient amount of time^32–36^. In other words, this mechanism allows young organisms to learn the main visual features of social partners. However, in the natural environment the chicks must recognize their mother hen and siblings from different visual perspectives, regardless of the angle they are observed from. Since different views of social partners likely follow one another over short periods of time, chicks will have to lump them together to build a complete representation of their social companions^37^. A sensitivity to the probabilistic structure of images that are rapidly alternated might support this function, allowing chicks to generate a coherent representation of the imprinting object. Indeed, chicks seem to be motivated to acquire exposure to different views of the imprinting object, which would support the development of a comprehensive representation of the mother hen. This need may be at the basis of the tendency of chicks to express, under some circumstances, a preference for slight novelty after imprinting. Similar tendencies have been observed already in the seminal studies of Patrick Bateson (e.g.,^38^) and are believed to help chicks to acquire information on the appearance of the imprinting object when observed from different perspectives. Various factors seem to modulate the direction of chicks’ preferences, for familiarity or novelty, such as the duration of exposure to the imprinting stimulus and the degree of difference between the familiar and the novel stimulus^38^. Intriguingly, sex is one of the main factors that affect chicks’ propensity for approaching slightly novel stimuli. A large literature documents sex differences in the *direction* of preference after imprinting, with male chicks preferring novel stimuli and females familiar ones^39–46^. Surprisingly, in our original work on statistical learning we did not observe the expected difference between the sexes (^28^, Experiment 1). In fact, both male and female chicks showed an identical preference for the unfamiliar sequence of shapes (i.e., for the sequence where the familiar shapes were presented in a semi-randomised order). The chicks tested in our original study were of the Hybro strain (a local strain derived from the white Leghorn breed). However, other chicken strains might indeed present the expected pattern of sex differences^45,47–49^.

**Figure 1:**
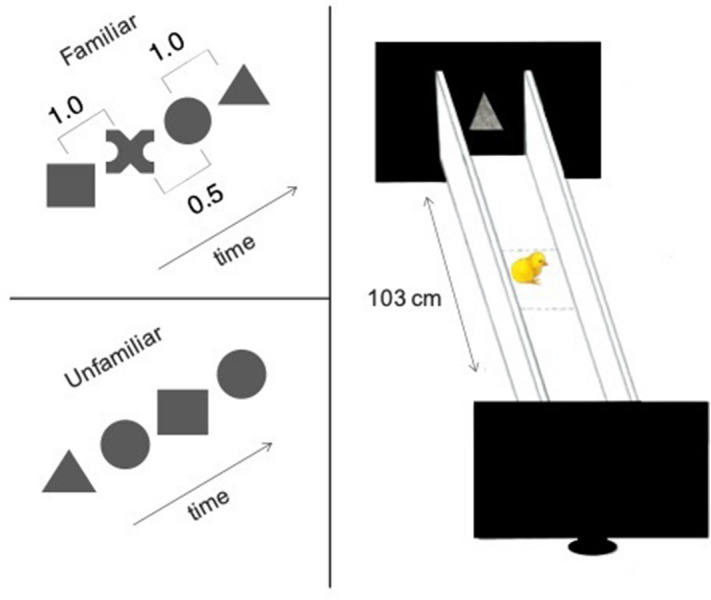
Illustration of stimuli and apparatus used in the experiment. *Top left*: example of the familiar structured sequence used at test. *Bottom left*: example of the unfamiliar random sequence. *Right*: schematic drawing of apparatus and computer screens used at test.

The aim of the present research is thus to explore how sex differences in the direction of preference (for familiarity or novelty) may impact results in a visual statistical learning task. We re-ran Experiment 1 of our original paper^28^ in a different chicken strain, the Aviagen Ross 308. This strain is the golden standard for broiler chickens, in which sex differences have been already reported in similar tasks^27^. We employed our standard imprinting protocol, according to which newly-hatched chicks undergo 2-h exposure to a visual stimulus, followed by a resting period in darkness, and a 6-min free-choice test between the imprinting stimulus and a novel. Stimuli differed solely in their statistical structure (Fig. 1). Chicks had no visual experience prior to the beginning of the experiment and were tested individually in a longitudinal choice corridor (Fig. 1) within a sound-attenuated room.

## Results

**Figure 2:**
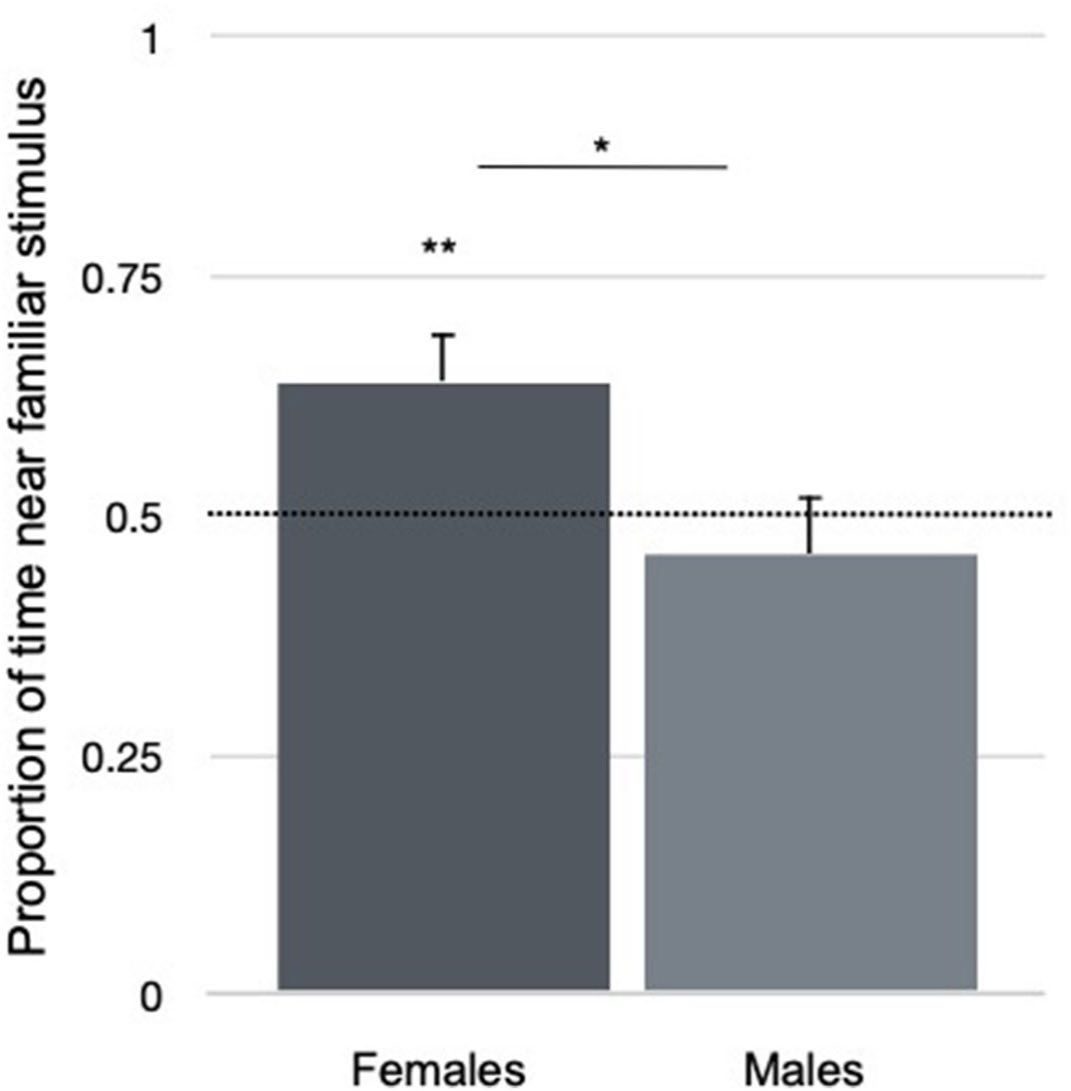
Mean proportion of time spent by female and male chicks near the screen presenting the familiar stimulus. Error bars show standard errors of the means. Asterisks indicate significance deviation from chance (** < 0.01), and significant comparison between groups (* < 0.05).

An Independent-Samples t-Test revealed a significant difference between females and males in the proportion of time spent near the familiar sequence over the total choice time (t_126_ = − 2.332, *p* = 0.021, 95% CI = [− 0.343, − 0.028], *d* = 0.412, Fig. 2). Two-tailed One-Sample t-Tests (against chance level, 0.5) were run separately for the two sexes. While females stayed significantly longer near the familiar sequence (mean proportion of time = 0.64; t_63_ = 2.784, *p* = 0.007, SEM = 0.052, *d* = 0.34, 95% CI [0.041, 0.252]), males did not show a clear preference, even though they tended to spend more time near the novel sequence (mean = 0.46; t_63_ = − 0.648, *p* = 0.519, SEM = 0.059, 95% CI [− 0.157, 0.08]) (Fig. 2). We also analyzed the first stimulus approached by each animal during the test. For this dependent variable, no cross-sex differences (X^2^_(1, 128)_ = 3.858, *p* = 0.074), nor significant first preference for any of the test sequences (X^2^_(1,128)_ = 2.531, *p* = 0.133) appeared. These results show that female Ross 308 chicks discriminated the familiar structured sequence from the unfamiliar random one, replicating our original findings in *Hybro* chicks (a different, local strain derived from *White Leghorns*). Chicks’ preferences were also scored offline using an automated tracking of the animals’ movements (see Methods). Manual and automated coding methods showed an extremely high correlation (*r*_117_ = 0.97, *p* < 0.001).

## General discussion

The results confirm newborn chicks’ sensitivity to the statistical structure of visual sequential inputs, mirroring the findings of the study by Santolin et al.^28^ at least in female chicks. After mere unrewarded exposure to a structured sequence of shapes, female chicks were able to discriminate between the imprinting sequence and a semi-random presentation of the same familiar shapes. As in^28^, this could be achieved only by encoding either the co-occurrence frequencies of the sequence elements (i.e., how often two shapes appear together), or the predictive relations within pair elements (i.e., the transitional probabilities between the two shapes)^50^. Overall, these results are consistent with previous infant research^6,9^ as well as studies on chicks and other species (e.g.,^22,24–27^) highlighting the importance of imprinting research for the uncovering of animals’ spontaneous abstraction abilities.

Importantly, our evidence confirms the pivotal role of sex in determining familiarity or novelty preferences in this species, and its interaction with the chicks’ strain. The comparison between our current results and those of Santolin et al.^28^ shows that the impact of sex in the direction of chicks’ preferences can vary between strains (White Leghorn *Hybro* and Aviagen Ross 308). While *Hybro* chicks did not display any sex difference and showed a preference for novelty common to both sexes, Aviagen Ross 308 chicks displayed significant sex differences. Females revealed a significant preference for the familiar stimulus, which was not apparent in any of the two sexes in the other breed, while males tended to approach more the novel stimulus. In this sense, the behavior of the breed that we employed in the current study is more in accordance with the existing literature and with our initial expectations on the direction of preference in the two sexes^27,43,44^. Importantly, we believe that the lack of preference in males of the current strain unlikely reflects lack of discrimination. Compared to the strain originally employed by Santolin et al.^28^, Aviagen Ross 308 chicks seem to exhibit stronger affiliation to familiar objects. This is congruent with females choosing familiar, and males alternating their choices between familiar or novel (as we said, there is indeed a trend for choosing novelty, as shown for males in other studies involving less subtle perceptual discrimination^27^).

Our results clearly highlight the importance of sex dimorphisms in behavioral experiments with domestic chicks, which needs to be contextualized in the general ecology and adaptation of this species. Overall, early social behavior has been shown to diverge between males and females. The two sexes seem to differ in their motivation to social reinstatement, with females displaying the higher motivation to keep contact with social companions. For example, females tend to maintain closer contact with the mother hen than males^51^. Likewise, while females are more motivated to expend an effort to reach social companions, males will more readily perform tasks for food rewards^40^ and are more aggressive towards social companions^39^. In female chicks, motivation to reach social companions, may even interfere with fear anti-predation responses, which would then appear to be more pronounced in males^52,53^. Notably, studies on imprinting on point-light displays of biological motion, revealed a complex interplay between sex and breed in determining the observed effects^43,46^, in line with what we have reported in the current study. In a study run with chicks of the Juila Leghorn strain^46^, males seemed to show a more “general” predisposition for biological motion, while females showed more “specific” predispositions, facilitating imprinting towards their species-specific walking pattern. However, using Hybro chicks, Regolin et al.^43^ revealed a different pattern of results: when stimuli also differed in their local motion properties, both sexes showed a preference for the novel stimulus (as in our previous work^28^); on the contrary, when stimuli differed in their global motion configuration, females approached the familiar imprinting animation, while males approached the novel one (as often reported in imprinting studies).

The presence of sex differences in this strain of chicks provides a plausible hypothesis for the absence of significant discrimination reported in a recent statistical learning study by Wood et al.^54^, which did not distinguish between male and female chicks. Pooling sexes together could have compromised the results across sample, reducing the effect to chance level, as suggested by a large literature on sex differences in the preference for familiar vs. novel objects (e.g.,^43–46^). Moreover, existing evidence indicate that chicken strains can differ as to the direction of their social preferences (also modulated by sex): some strains being more prone to explore novel objects than others^45,47–49^. Our results are consistent with this literature, showing that various strains may present different patterns of sex dimorphism in the direction of their preference. This makes it unadvisable to employ non-auto sexing breeds, such as the Rhode Island Red used by Wood and collaborators, for which it is nearly impossible to determine the sex of each animal from its external appearance. However, one could argue that if sex dimorphism is the reason behind the absence of preference reported in^54^, individual data should show a bimodal distribution, with half of the animals preferring the familiar and half the novel stimulus. Unfortunately, individual data reported by Wood et al.^54^ refer only to the whole test duration in which each chick’s preference was measured over repeated cycles of 20-min testing. In contrast, the very first minutes of test are crucial and therefore used as standard window to measure preferences in chicks. This raises the additional issue of using long and repeated testing sessions in imprinting procedures, a process affected by sensitive periods^55^. Since preferential choices are expected to appear within the very first minutes of test, repeated testing of the same animal over a much longer time would likely mask any initial meaningful preference. Moreover, repeated testing may have led to *secondary imprinting* (according to which chicks imprint on the previously novel stimulus) contaminating further testing (see^56–58^).

Non-significant results are notoriously difficult to interpret, hence it is difficult to determine with certainty the reason of the non-significant effects reported in Wood et al.^54^, especially when other multiple factors could have compromised discrimination, such as small sample size and lack of control over the acoustical environment (due to simultaneous testing of many chicks in adjacent cages). Conspecifics’ calls work as effectively as visual stimulation in modulating filial imprinting, acting as a powerful (but uncontrolled) cue in directing chicks’ attention at test^32,59^. Note that sustained attention to the stimuli is essential in statistical learning to track the sequence of transitions, whereas it is immaterial for much easier tasks such as recovering the mere shape composing the stimuli (which Wood et al.^54^ chicks were indeed capable to grasp). Future research should be devoted to clarifying the role of similar methodological factors in determining performance in imprinting-based statistical learning task (see in this regard also Lemaire^60^).

Going back to our original question, what could be the ecological meaning of the prominent sex effects emerging in the literature? The differences in early social behaviour in male and female chicks may reflect specific adaptations to the adult social and territorial behaviour of the two sexes. In the natural environment, a feral cock will control a large territory, simultaneously inhabited by several females^61^. Indeed, when reaching adulthood, male chicks need to disperse away from their native home range, leaving their social group of origin. The territorial behaviour of feral chickens may thus promote stronger sociality in females and aggressive or exploratory tendencies in males.

The current research paves the way to further investigation of the adaptive function of statistical learning in newborn domestic chicks. Along with previous results^28^, we have shown here that filial imprinting and visual statistical learning are likely to work in tandem to allow chicks to develop a comprehensive representation of social companions. Indeed, tracking the ordering of a sequential visual input might be adaptive for chicks to integrate the different sides of the social imprinting object seen from different perspectives. It is crucial to take into consideration the biology of the species when designing experiments and interpreting results. For chicks, sex and strain appear to be fundamental factors to consider in order to recognize the ecological function of the behaviors manifested at test.

## Methods

### Ethical statement

In compliance with Italian and European Regulations all the experiments and the experimental procedures reported in this paper were approved by the OPBA (“Organismo Preposto al Benessere Animale”, committee for animal welfare) of the University of Trento. In addition, all procedures and experiments were evaluated by the Italian Istituto Superiore di Sanità (Italian National Institute of Health) and explicitly approved and licensed by the “Ministero della Salute, Dipartimento Alimenti, Nutrizione e Sanità Pubblica Veterinaria” (Italian Ministry of Health, Department for Aliments, Nutrition and Public and Veterinary Health). The permit number assigned to these experiments by the Italian Ministry of Health is 987/2017. All methods of the experiment were performed in accordance with relevant guidelines and regulations.

### Subjects

We tested 128 (64 males) domestic chicks (*G. gallus*, Aviagen Ross 308). Fertilized eggs were obtained from a commercial hatchery (CRESCENTI Società Agricola S.r.l.—Allevamento Trepola—cod. Allevamento127BS105/2, Italy). With respect to the original paper, experiments were carried out in Trento (GV’s labs) rather than in Padova (LR). On arrival, eggs were placed in an incubator (FIEM, MG 200/300 super rural) until day 19 of incubation. Standard conditions for optimal incubation were provided (temperature 37.7 °C; humidity 40%). From day 19, the eggs were moved to a hatchery (FIEM, MG 140/200 rurale) with the same temperature as the incubator, but at a higher humidity (60%). The incubator, the hatchery and the hatching room were maintained in complete darkness. Behavioral observations took place on the first day after hatching (which takes place on day 21 of incubation). At the end of the observations, chicks were donated to local farmers. Chicks’ sex was determined immediately prior to exposure to the imprinting stimulus, by rapid inspection of the wing feathers of each bird (this is a breed of auto-sexing animals). Feather-sexing was performed by expert experimenters; it required only few seconds for each chick, the animal’s head was covered and the procedure took place in semi-darkness.

### Stimuli and apparatus

We used identical stimuli and apparatus to those used in Exp. 1 of Santolin et al.^28^. The exposure sequence consisted of two shape-pairs defined by statistical dependencies within and between pairs. Each shape was presented for 2 s, looming from 2 to 10 cm in height on an Asus MG248QR 24 monitor. The familiarization sequence was composed of orange shapes. Pair 1 consisted of a square always followed by a cross, with a transitional probability (TP) of 1, and Pair 2 consisted of a circle always followed by a triangle (TP = 1). The element that appeared after each pair was the first item of one of the two pairs (TP between-pairs = 0.5). Repetitions of the same pair were allowed, as in prior statistical learning studies and in our original work. As in the original study^28^, there were no temporal delays (pauses) between shapes and shape-pairs therefore the only available cue to segment the sequence was its statistical structure. During imprinting, which lasted 2 h, each shape-pair appeared 1,800 times whereas at test (whose duration was 6 min) each shape-pair appeared 90 times.

Test stimuli consisted of the exposure (familiar) sequence, and an unfamiliar sequence, which was a semi-random generation of the same four items (with the constraint that two identical items could never appear consecutively). Importantly, the only difference between familiar and unfamiliar test sequence was the statistical structure of the elements. Shapes’ color was removed at test (using neutral grey), as in the original experiment, to prevent color saliency to override potential discrimination.

In order to test the role of screens’ refresh rate and of stimuli’s frame rate, we initially created two conditions manipulating these factors. Half of the chicks (64 animals, 32 M) were imprinted and tested using higher refresh rate and number of frames per seconds (60 Hz and 60 fps, as in our original study^28^). The other half of the sample (64 chicks, 32 M) were imprinted and tested setting the screens at a 24 Hz refresh rate and using stimuli with 24 fps, similarly to what was done in the work of Wood et al.^54^. However, this manipulation did not have any significant effect on chicks’ preference between the two stimuli (F_(1,124)_ = 0.432, *p* = 0.512), nor it did interact with chicks’ sex (F_(1,124)_ = 0.157, *p* = 0.693, 24 fps males = 0.50 ± 0.084, mean ± SEM; 24 fps females = 0.66 ± 0.074; 60 fps males = 0.42 ± 0.084; 60 fps females = 0.64 ± 0.076). For this reason, we have collapsed these two conditions for all further analyses. Future studies may be specifically devoted to further investigate the effect of this manipulation.

The test apparatus was a white plywood runway (20 × 103 × 30 cm), divided into a central sector (15 cm) and two lateral sectors (each one of 44 cm). Two identical computer screens (Asus MG248QR 24) were placed at the opposite ends of the runway, simultaneously playing the two test sequences. Apart from the light arising from the monitor, the room was kept in complete darkness and silence.

### Procedure

Exposure and testing were carried in the exact same way as in Exp. 1 of Santolin et al.^28^. On the first day of life, chicks were taken from the dark hatchery in a closed cardboard box, and placed individually in a cubicle (10 × 10 cm) from which they were exposed to the familiar stimulus for 120 consecutive minutes (familiarization phase) at a viewing distance of 50 cm. At the end of the exposure time, each chick was taken from its cubicle and placed in another hatchery, completely empty and dark (so chicks could not see each other), for 30 min. Importantly, chicks could *not* see one another at any time during familiarization or resting phase.

Afterwards, each chick was tested individually in an isolated and sound-attenuated room. At the beginning of the test, the chick was placed in the central area of the apparatus (runway), facing either one of the two long sides. The screen playing the familiar sequence could be either the one to the right or to the left side of the apparatus. These two factors were counterbalanced between subjects. Chicks’ movements along the runway were observed for 6 consecutive minutes and recorded by a webcam placed on top of the apparatus. Entrance and permanence of the chick in one of the sectors were considered as a preference for the stimulus presented at that end. These measures were scored on-line by an experimenter (blind to the hypotheses being tested) who observed the animals through a TV-screen connected to the webcam, as in^28^. Whenever behaviour was considered potentially ambiguous to score, test was re-scored offline by a second coder, blind to stimuli position and sex of the chick. In this case, the offline blind coding only was used for the analyses, to maximize data objectivity. Manual coding was compared to coding performed by an automated tracking based on *Visual Field Analysis*, an application for the use of the DeepLabCut system^62^. This is a Python-based tracking toolbox that uses deep learning techniques to track animals’ movements with great accuracy^63,64^. Also in this case, all video processing was performed blind to chicks’ sex, stimuli position, and the hypotheses under test. We were able to successfully track 119 out of 128 videos, obtaining an extremely high correlation between the manual and the automated coding (Pearson’s correlation = 0.965, *p* < 0.001). This also confirms the reliability of the standard coding techniques employed in our laboratories.

### Statistical analysis

Sample size was determined prior to data collection by a power analysis (G*Power, Version 3.1). The main aim of the study was to document the presence of sex differences in the direction of preference thus, the power analysis was run for a Two-Tailed Independent-Samples t-Test comparing females and males. Since the original study was ran on a different strain showing no sex differences for this specific test, we assumed a Cohen’s *d* of 0.5 (conventionally considered as medium) for the current study. To reach 80% power, 128 chicks were tested (64 per each sex group). The main dependent variable was *Proportion of time* spent near the familiar sequence, and was coded as follows: [*time spent by familiar stimulus*/(*time spent by familiar stimulus* + *time spent by unfamiliar stimulus*)], as in Santolin et al.^28^. In addition to such measure, we analyzed the *First stimulus approached* by the animals with a Chi-Square test applied on a 2 × 2 contingency table, as in the original experiment.

## Data Availability

The datasets generated during and/or analysed during the current study are available in the OSF repository, https://osf.io/vsnfb/.
